# Conversion disorder: an inclusive approach based on its history

**DOI:** 10.1192/j.eurpsy.2025.1244

**Published:** 2025-08-26

**Authors:** M. M. Marques

**Affiliations:** 1 Psychiatry, ULSAM, Viana do Castelo, Portugal

## Abstract

**Introduction:**

The conversion symptom, present in current medical practice, traces its origins to Ancient Egypt, having had, over time, several interpretations. Under the name Hysteria, it was studied by Charcot with hypnosis at the Salpêtrière Hospital. Currently, it belongs to Conversion Disorders, and neurology has given the term psychogenic nonepileptic seizures (PNES), to differentiate these phenomena from epileptic seizures.

**Objectives:**

This work proposes to describe the history of Conversion disorder, from Freud’s hysteria to the concept of psychogenic nonepileptic seizures, and to summarize useful concepts in approaching patients with conversion disorder, in different contexts.

**Methods:**

Non-systematic review of the literature with selection of scientific articles, using PUBMED as database the following keywords: «Conversion disorder», «hysteria» and « psychogenic nonepileptic seizures». Seven articles were included. We also selected 5 reference books.

**Results:**

In the 19th century, the conversion symptom constituted a very important milestone in Psychiatry, as it was through monitoring patients with hysteria that Freud created Psychoanalysis. Then, the physical symptoms of hysteria (paralysis, convulsions…) began to be understood as symptoms of psychic origin, and as a symbolic expression of a representation that was unacceptable to the Self and, therefore, repressed. According to psychoanalysis, in hysteria, body is invaded by psychic and serves as a stage for unconscious representations. Nowadays, the term hysteria can include psychogenic nonepileptic seizures, which are paroxysmal episodes of changes in behavior, movement, sensitivity or consciousness, similar to epileptic seizures, but which, unlike epileptic seizures, are not caused by a change in brain’s electrical activity.

**Conclusions:**

With the description of the evolution of conversion disorder, from Freud’s hysteria to nowadays, it is possible to find that conversion pathology will not tend to disappear. It represents a specific mode of psychic functioning, characterized by an organization of defense mechanisms, cognitive styles, memory functions and specific personality traits. Therefore, it is important to continue investigating strategies to individualize the approach to these patients. Also, it would be beneficial to extend access to important psychological knowledge to more general doctors, in order to improve the capacity of managing conversion disorders cases that arise in different health contexts. We highlight the importance of understanding the suffering of these people. Patients with conversion symptoms do not choose to have a certain symptom and shouldn’t be a target of stigma. Each person develops their own psychic functioning process, as each person constitutes an unique identity and deserves an individualized and inclusive approach.

**Disclosure of Interest:**

None Declared

